# Retraction:

**DOI:** 10.1002/jia2.26068

**Published:** 2023-02-27

Adhikari, R. (2010), Are Nepali students at risk of HIV? A cross‐sectional study of condom use at first sexual intercourse among college students in Kathmandu. *Journal of the International AIDS Society*, 13: 7–7. https://doi.org/10.1186/1758-2652-13-7

“Are Nepali students at risk of HIV? A cross‐sectional study of condom use at first sexual intercourse among college students in Kathmandu” (the “Article”) by Ramesh Adhikari (the “Author”) in the Journal of the International AIDS Society (the “Journal”), published online on 2 March 2010 on Wiley Online Library (wileyonlinelibrary.com), has been retracted by agreement between the Journal's Editors, the International AIDS Society and John Wiley & Sons Ltd. This retraction has been agreed as the author did not obtain the necessary ethical approval from the Nepal Health Research Council.

